# Safety performance behaviors of hospital nurses from the perspective of social cognition theory

**DOI:** 10.3389/fpsyg.2025.1676200

**Published:** 2025-11-28

**Authors:** Changde Chi, Guomei Gong, Xiaoling Zhang, Xuehua Cai, Junxia Chen, Hanying Jiang

**Affiliations:** 1 School of Nursing, Quanzhou Medical College, Quanzhou, China; 2 Department of Nursing, Quanzhou First Hospital, Quanzhou, China

**Keywords:** safety performance, nurses’ safety behaviors, social cognitive theory, anticipation orientation, structural equation modeling (SEM)

## Abstract

**Objectives:**

This study aimed to investigate the safety performance behaviors of hospital nurses by developing and testing a comprehensive model based on social cognitive theory (SCT).

**Methods:**

A cross-sectional, survey-based study was conducted. Data were collected from 269 registered nurses across multiple hospitals in Quanzhou, China, via an online questionnaire. Previously validated scales were adapted to measure the constructs. The proposed research model and hypotheses were tested using partial least squares structural equation modeling (PLS-SEM).

**Results:**

The results showed that safety climate had a significant positive effect on safety motivation (*β* = 0.716, p < 0.001). Safety motivation, in turn, positively influenced both safety compliance (*β* = 0.498, *p* < 0.001) and safety participation (*β* = 0.195, *p* < 0.01). Anticipation orientation mediated the relationship between safety motivation and both safety behaviors. Psychological ownership for safety promotion was a strong predictor of anticipation orientation (*β* = 0.537, *p* < 0.001). Furthermore, co-worker support positively moderated the relationship between safety motivation and safety participation (*β* = 0.220, *p* < 0.01) but did not have a significant moderating effect on the motivation-compliance relationship.

**Conclusions:**

The findings underscore the importance of a multi-faceted approach to enhancing nurse safety performance. Healthcare institutions should prioritize fostering a positive safety climate, cultivating nurses’ psychological ownership of safety, and strengthening co-worker support networks to effectively translate motivation into proactive safety behaviors. This study advances the application of SCT in nursing safety by integrating organizational, cognitive, and social factors into a unified framework.

## 1 Introduction

Safety performance in hospital environments is crucial for patient safety and healthcare quality, with nurses’ safety performance behaviors directly influencing medical service safety and efficiency as they are vital healthcare team members (Iyasere et al., 2022). In recent years, with the increasing exposure of healthcare safety incidents, there has been heightened academic focus on the safety performance of healthcare professionals, particularly nurses (Kupkovicova et al., 2024; Leon et al., 2024). Previous research has applied SCT (Bandura, 1986) to other than nursing workplace safety, these studies often focus on a limited set of factors—typically organizational climate or individual cognition—in isolation, thereby offering a fragmented view of the safety behavior ecosystem. This study advances the existing SCT-based safety literature by proposing and empirically testing a more comprehensive framework that simultaneously incorporates and articulates the interplay between three core elements of SCT: organizational influences (safety climate), cognitive-psychological factors (psychological ownership for safety promotion and safety motivation), and social supports (co-worker support).

The concept of workplace climate—an essential component of hospital organizational culture (Falcone et al., 2023)—has been examined in contexts such as service (Lee et al., 2021), ethics (Morgan et al., 2024), and innovation (Akkoç et al., 2022). Emerging evidence suggests that leadership plays a pivotal role in shaping safety climate; specifically, transformational leadership has been shown to strengthen patient safety culture, which in turn enhances safety practices among nurses (Hamdan et al., 2024). This underscores the importance of examining safety climate as a central relevant within our theoretical model. However, there is a lack of literature research on the specific mechanisms of workplace safety atmosphere in promoting nurse safety behaviors. At present, most of the literature on nurse safety behaviors research does not distinguish between the two dimensions of nurse safety performance behaviors: safety compliance and safety participation. The research model is significant in that it distinguishes between two dimensions of nurses’ safety performance behaviors: safety compliance (e.g., adherence to safety protocols) and safety participation (e.g., voluntary involvement in safety-enhancing activities). It is critical for healthcare organizations to promote nurses’ safety-related task performance and contextual performance (Mashi et al., 2023). Compliance with safety protocols, including hand hygiene, proper use of personal protective equipment (PPE), and adherence to infection control guidelines, is a critical aspect of nursing practice (Kim et al., 2021). Contextual performance, particularly engagement and active participation, refers to nurses’ proactive involvement in the broader safety culture within the hospital, which includes taking part in safety training, suggesting improvements in safety practices, engaging in team safety discussions, and contributing to the overall development of safety protocols (Kim et al., 2021).

While the influence of organizational culture and leadership on safety performance in healthcare has been extensively studied, there remains a limited focus on individual-level factors, such as safety ownership, that could directly shape safety behaviors (Gomes et al., 2024). For example, few previous studies have explored how a sense of personal ownership over safety directly impacts safety prevention orientation, which refers to the proactive behaviors aimed at preventing safety hazards before they occur (Curcuruto et al., 2016). In addition, much of the research on safety ownership has concentrated on industrial and manufacturing settings, with comparatively little attention given to the healthcare context (Curcuruto et al., 2016). Given the unique, high-risk challenges in hospitals demanding nurses’ proactive safety efforts, this study aims to bridge the gap by exploring how safety ownership impacts nurses’ safety prevention behaviors.

Coworker support is vital in healthcare. Nurses, working in teams under high pressure, depend on each other emotionally and practically. While some studies have identified the influence of coworker support on safety outcomes, they often focus on general work performance or individual-level motivation (Wang and Tang, 2022). There is still insufficient research on the unique regulatory mechanism between coworker support and safety motivation and safety performance behaviors. Past studies have predominantly focused on the direct relationship between safety motivation and safety behaviors (Huang et al., 2024). However, the role of psychological mediators, particularly safety prevention orientation, has received less attention. Although a few studies have explored similar mediating constructs, such as safety climate and safety knowledge (Neal and Griffin, 2006), few have investigated how safety prevention orientation specifically mediates the relationship between safety motivation and safety behaviors in the context of nursing.

This study introduces and validates an SCT based framework that addresses three research gaps in nursing safety: (1) the role of organizational safety climate in driving safety motivation has not been fully explored; (2) Individual: Neglected impact of safety psychological ownership (nurse’s personal responsibility for safety) as a predictor of positive safety behaviors; (3) Society: The impact of insufficient theoretical support from colleagues in strengthening the connection between motivation and behaviors. This framework uniquely distinguishes between safety compliance (compliance with protocols) and participation (active participation), reflecting the dual requirements of healthcare for specific tasks and situational safety performance. Empirically speaking, it tested how safety motivation regulates climate effects and how expectation orientation relates motivation to behavioral translation. To our knowledge, this represents the first SCT application in nursing safety that not only integrates organizational, individual, and social factors but also explicitly differentiates the pathways leading to compliance versus participation, thereby providing a more nuanced and mechanism-rich understanding of nurse safety behaviors and extending the explanatory power of SCT in high-reliability healthcare settings.

## 2 Research model and hypotheses development

### 2.1 Social cognitive theory and nurse safety performance behaviors

Social Cognitive Theory (SCT), originally proposed provides a coherent framework for comprehending safety behaviors among hospital nurses through its emphasis on the triadic reciprocity among cognitive, behavioral, and environmental factors. It suggests that individuals learn from direct experiences, as well as by observing others and interpreting behavior outcomes to form cognitive representations guiding future actions. In nursing safety, three key SCT mechanisms are relevant: observational learning, where nurses learn safety practices by observing peers and leaders; self-efficacy, which boosts motivation to follow protocols through confidence in performing safety procedures; and reciprocal determinism, where personal factors, behaviors, and environmental cues continuously interact to shape safety performance. Thus, SCT offers a theoretically grounded perspective on how nurses’ work environment perceptions, personal safety beliefs, and social influences collectively shape safety-related behaviors.

### 2.2 The direct effect of safety climate on safety motivation

Safety climate refers to an individual’s subjective perception and understanding of the safety culture and safety practices within their work environment. This perception includes awareness of workplace safety policies, the support and communication among colleagues, and the level of commitment to safety shown by management. While there is no universally accepted definition of safety climate, management’s support and commitment to safety are generally considered the core elements in building a safety climate. When hospital management places a high emphasis on health and safety issues in the workplace, nurses and other healthcare workers tend to perceive a stronger safety climate. Safety motivation is defined as the psychological drive that influences individuals to engage in safety-related behaviors and actions to avoid harm or mitigate risks (Neal and Griffin, 2006). Huang et al. (2024) conceptualize safety motivation as an individual’s cognitive and emotional willingness to engage in behaviors that reduce the likelihood of injury and promote a safety-conscious culture, with a focus on the role of both organizational policies and personal attitudes.

According to SCT, one of its core concepts is self-efficacy, which refers to an individual’s confidence in their ability to successfully complete a specific task. Safety climate, as a social environmental factor, positively influences employees’ safety motivation by enhancing their self-efficacy. Furthermore, Bernardes et al. (2025) highlights that the safety climate within an organization influences employees’ perceptions of risk and safety norms, which in turn affects their motivation to engage in safety-related activities. Therefore, we propose the following hypothesis:

*H1*: Safety climate is positively correlated with safety motivation.

### 2.3 Safety performance behaviors: safety compliance and safety participation

The classical definition of safety performance behaviors has been foundational in understanding how workers engage with safety protocols and procedures. According to (Neal and Griffin, 2006), safety performance behaviors can be categorized into two distinct dimensions: safety compliance and safety participation. Safety compliance refers to employees’ adherence to organizational safety protocols, such as using protective equipment, following procedures, and adhering to safety regulations. Safety participation involves voluntary behaviors beyond the minimum compliance, such as proactively suggesting improvements to safety processes, participating in safety committees, or helping colleagues with safety concerns. Importantly, contemporary research has expanded the conceptualization of workplace determinants influencing these behaviors beyond the social-organizational climate. For instance, the physical work environment—including factors such as workspace design, equipment availability, and ergonomic conditions—has been shown to significantly impact nurses’ safety compliance through serial psychological and behavioral pathways (Al-Bsheish et al., 2023).

According to SCT, motivation arises from individuals’ beliefs in their ability to perform specific behaviors, and this belief (self-efficacy) drives actions, such as adhering to safety protocols (safety compliance) or engaging in proactive safety behaviors (safety participation). Recent studies have supported this view, highlighting that motivated nurses are more likely to comply with safety regulations and actively participate in safety initiatives. For instance, Seo and Lee (2022) found that nurses with higher safety motivation demonstrated greater adherence to safety standards and actively contributed to safety audits and discussions. Similarly, Huang et al. (2024) confirmed that safety motivation significantly enhances both safety compliance and safety participation among healthcare workers. Based on this logic, we propose the following hypotheses:

*H2a*: Safety motivation is positively related to safety compliance.

*H2b*: Safety motivation is positively related to safety participation.

### 2.4 The moderating role of co-worker support

The original and classic definition of “co-worker support” can be traced back to the work of House (1981), who conceptualized social support as “the emotional, informational, and instrumental assistance provided by individuals in one’s social network.” In this context, co-worker support refers specifically to the assistance and resources provided by colleagues in the workplace. It includes various forms of help, ranging from emotional encouragement to practical help, such as sharing workload or providing advice in problem-solving situations. Syed-Yahya et al. (2023) study showed that strong social support from co-workers enhances safety motivation by fostering a collaborative environment where individuals feel more responsible for maintaining safe work behaviors. Similarly, Syed-Yahya et al. (2022) found that when co-worker support is high, employees are more likely to be motivated to engage in safety practices because they perceive safety as a collective responsibility, thereby increasing their safety motivation. According to SCT, behavior is influenced by social interactions within one’s environment, In the workplace, individuals are more likely to engage in safe behaviors when they are motivated to do so and when they perceive that their peers support such behaviors. As such, we hypothesize the following hypotheses:

*H3a*: Co-worker support positively moderates the relationship between safety motivation and safety compliance, such that the positive impact of safety motivation on safety compliance is strengthened (weakened)when the level of Co-worker support is high (low).

*H3b*: Co-worker support positively moderates the relationship between safety motivation and safety participation, such that the positive impact of safety motivation on safety participation, is strengthened (weakened)when the level of Co-worker support is high (low).

### 2.5 The mediating effects of anticipation orientation

Anticipation orientation is a mindset and behavioral pattern oriented towards the future, emphasizing individuals’ proactive prediction of potential safety risks and uncertainties, followed by the implementation of measures to avoid and mitigate these risks before they occur (Curcuruto et al., 2016). When nurses possess such a predictive mindset, they can maintain heightened sensitivity to potential safety hazards in their work at all times (Atalla et al., 2024). Although existing research on safety has shown a direct link between safety motivation and safety performance, anticipation orientation may serve as a mediating factor in this relationship.

Several studies have explored the influence of safety motivation on anticipation orientation in various work settings. Sharma et al. (2025) focused on the transportation sector, finding that safety motivation has a significant impact on anticipation orientation. Anticipation orientation also plays a key role in influencing safety performance behaviors, such as safety compliance and safety participation. In industries such as construction and manufacturing, Curcuruto et al. (2016) found that employees with higher anticipation orientation were more likely to comply with safety regulations and actively participate in safety programs. Building on SCT, Safety motivation, as an intrinsic driver, influences cognitive processes such as anticipation orientation, which, in turn, facilitates safer behaviors. Thus, we propose the following mediation hypotheses:

*H4a*: Anticipation orientation mediates the relationship between safety motivation and safety compliance.

*H4b*: Anticipation orientation mediates the relationship between safety motivation and safety participation.

### 2.6 The direct effect of psychological ownership for safety promotion on anticipation orientation

Psychological ownership for safety promotion refers to the emotional and cognitive feeling of ownership that employees experience regarding workplace safety, leading them to take personal responsibility for maintaining and improving safety conditions (Curcuruto et al., 2016). This concept involves employees perceiving themselves as integral participants in the safety process, feeling that they have a stake in the safe operation of the organization, and acting proactively to enhance safety behaviors and culture (Curcuruto et al., 2016).

SCT suggests that individuals’ behaviors are influenced by their cognitive processes, including how they anticipate future outcomes based on their actions. When employees experience psychological ownership for safety, they are more likely to view safety as a personal responsibility, and this heightened sense of ownership can lead them to anticipate and plan for potential safety hazards before they occur. According to research on psychological ownership (Novieto, 2023), when individuals feel ownership over an aspect of their work, they tend to be more proactive in managing risks, as they perceive themselves as key players in the safety system. This proactive mindset aligns with anticipation orientation—the tendency to focus on future challenges, identify potential problems, and take preventive measures (Curcuruto et al., 2016). Therefore, we propose that:

*H5*: psychological ownership for safety promotion is positively related to anticipation orientation.

**Figure 1:**
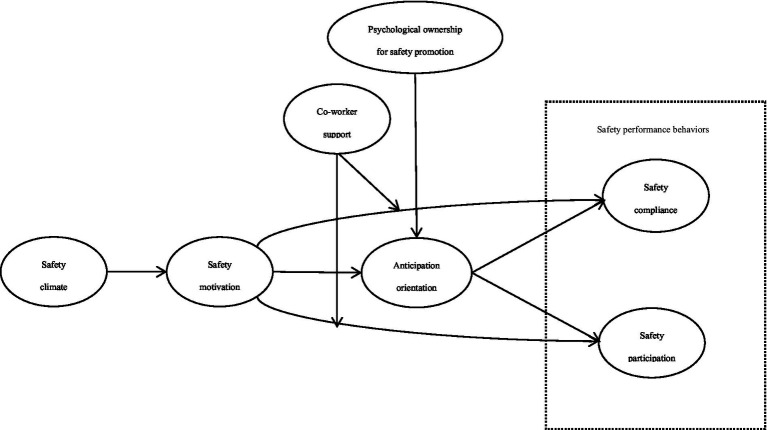
Research model.

The overall research model is presented in Figure 1.

## 3 Research methodology

### 3.1 Construct operationalization

To evaluate our proposed model, we employed a survey-based approach and created a questionnaire. In order to establish content validity, the items in the questionnaire were adapted from previously validated scales. We made certain adjustments to better align these items with the specific context of our study, particularly the nursing environment, by incorporating domain-specific terminology and scenarios. For example, the original safety motivation item “I feel that it is important to maintain safety at all times” was modified to “I believe it is important to maintain nursing safety at all times.” to reflect nursing-specific tasks. The measurement scales for safety climate and safety motivation were both adapted from Neal and Griffin (2006). The measurement scales for anticipation orientation was adapted from Curcuruto et al. (2016). For safety compliance and safety participation, we adapted the measures from Vinodkumar and Bhasi (2010). The measurement scales for psychological ownership for safety promotion was adapted from Curcuruto et al. (2016). Finally, co-worker support was measured using the items in Leung et al. (2016). All constructs were measured using a 7-point Likert scale, ranging from 1 (strongly disagree) to 7 (strongly agree).

Since the original measurement scales were in English, we employed a back-translation technique to translate the English versions into Chinese. We consulted with three experts from both English language and nursing backgrounds to review the questionnaire. Several items were revised or enhanced to improve clarity. In order to further ensure content validity beyond expert review and pilot testing, we conducted a systematic content validity assessment using the Content Validity Index (CVI). Three nursing experts rated the relevance of each item on a 4-point scale, and items with a CVI below 0.78 were refined or replaced. A pilot test involving 50 participants was conducted to evaluate the construct validity, leading to adjustments in items with factor loadings below 0.7. The final list of measurement items is provided in the Table A1.

### 3.2 Data collection

After obtaining ethical approval from the Quanzhou Medical College Ethical Review Committee (Approval No.: QZMC-2025-032), data were collected through the Wenjuanxing online survey platform.^1^ Participants received an anonymous, self-administered questionnaire. Prior to accessing the questionnaire, an online informed consent form was presented on the first page of the survey. This form elaborated on the study’s purpose, procedures, potential risks and benefits, and the measures taken to ensure data anonymity and confidentiality. It explicitly stated that participation was voluntary and that participants had the right to withdraw at any time without penalty. Only participants who selected the option indicating their agreement to participate (e.g., by clicking “I agree” or “I consent to participate”) were directed to the formal questionnaire. Participants adhered to strict confidentiality protocols during data collection. All data were anonymized and securely stored to ensure participant privacy. We employed a stratified random sampling strategy to ensure representativeness across the seven hospitals in Quanzhou, China. First, hospitals were stratified by level (e.g., tertiary vs. secondary) and size. Then, within each hospital, nurses were randomly selected based on inclusion criteria: (1) being a registered nurse with at least one year of clinical experience, (2) currently employed full-time in direct patient care roles, and (3) voluntarily agreeing to participate. Exclusion criteria included nurses in administrative or temporary positions, those on extended leave during the study period, and participants with incomplete or inconsistent responses. To achieve balanced representation, sample sizes were allocated proportionally to each hospital based on the total number of eligible nurses, ensuring that both large and small institutions contributed adequately to the final sample. We contacted the nursing departments of seven hospitals in Quanzhou, China, and sent the questionnaire via WeChat with a uniform explanation of the importance of hospital participation and consent. After obtaining approval, nurses from the nursing departments helped distribute the questionnaires. Nurses could complete the survey by clicking the WeChat link or scanning the QR code. Before the survey was distributed, the researchers provided specialized training for three investigators to ensure they could efficiently administer the survey and monitor the quality and response rate of the electronic questionnaires. If any issues arose, the investigators promptly communicated with the nurses at each hospital to resolve them. Additionally, to encourage participation, nurses who completed the survey received a 3-yuan WeChat red envelope as a reward. This token incentive was intended to acknowledge participants’ time and effort rather than disproportionately influence participation decisions. To mitigate potential selection bias, such as primarily attracting individuals particularly motivated by small incentives, the following measures were implemented: (1) the incentive was uniform and not advertised as the primary reason to participate; (2) survey completion and preliminary data review were required before the red envelope was issued, helping to ensure response quality.

We invited 310 nurses to participate in the survey and received 277 responses, with a response rate of 92.3%. Invalid questionnaires were discarded based on the following criteria: (1) questionnaires with contradictory or illogical answers; (2) respondents who gave the same answer to all questions; (3) respondents who completed the questionnaire in a very short time (e.g., less than 60 s). After removing the 8 invalid responses, we obtained 269 valid responses, yielding a final valid response rate of 89.7%. The required sample size was determined *a priori* using G*Power software (version 3.1.9.4). Based on a power of 0.95, a statistical significance of 95% (*α* = 0.05) (two-tailed), a large effect size (*f* ^2^ = 0.35), and 2 predictors, the power analysis indicated a minimum requirement of 48 participants (Faul et al., 2007). The final sample for this study comprised 269 participants, a number that substantially exceeds the calculated minimum, thereby enhancing the statistical power and reliability of the findings. Table 1 summarizes the demographic characteristics of the respondents. As detailed in Table 1, the collected demographic and work-related variables included gender, age, education level, hospital level, work area (e.g., ICU, internal medicine, emergency), and years of experience as a registered nurse. More granular variables such as shift work patterns, precise unit workload, and nurse-to-patient ratios were not captured in this study. These factors could provide valuable contextual insight into safety performance. We acknowledge that this study’s exclusive focus on Quanzhou, China, may limit the generalizability of its findings. Local cultural factors (e.g., collectivistic norms in Eastern societies) and healthcare system characteristics (e.g., hierarchical management, resource allocation) may uniquely influence nurses’ safety behaviors in this region. For example, safety compliance and participation could be shaped by collective accountability or institutional policies specific to the Chinese healthcare context. Caution is advised when applying these results elsewhere, and we encourage future research to examine these dynamics in diverse cultural and organizational settings to assess cross-cultural validity.

**Table 1:** Profiles of respondents (*N* = 269).

Respondents	Category	Count	%
Gender	Female	259	96.28%
	Male	10	3.72%
Age	≤30	150	55.76%
	31–40	109	40.52%
	≥41	10	3.72%
Education	Bachelor’s degree or above	95	35.32
	associate’s degree or below	174	64.68
Hospital level	Tertiary hospitals	131	48.70
	Secondary hospitals	86	31.97
	Primary hospitals	52	19.33
Work area	Ambulatory and ER	60	22.30
	Internal medicine	80	29.74
	General surgery	45	16.73
	Obstetrics and gynecology	31	11.52
	Pediatrics	25	9.29
	Operating room	14	5.20
	ICU	14	5.20
Time as registered nurses	≤5 years	95	35.32
	6-9 years	73	27.14
	≥10 years	101	37.55

As addressed in previous research, the non-response bias issue is mitigated through a time-trend extrapolation method, comparing early and late respondents to assess any potential bias (Armstrong and Overton, 1977). Chi-square tests were conducted to compare early (first-quartile) and late (fourth-quartile) respondents on characteristics such as gender, age, and education. No significant differences (*p* > 0.5) were found, indicating that non-response bias was not a significant concern.

## 4 Data analysis and results

In this study, we utilized Structural Equation Modeling (SEM) to test the proposed research model. Smart PLS 4.0 was chosen as the primary statistical tool for evaluating both the measurement and structural models for two main reasons. Firstly, the Partial Least Squares (PLS) method is more appropriate for our data, which does not follow a normal distribution, as opposed to covariance-based methods that assume normality (Chin, 1998). Secondly, PLS is particularly advantageous for studies with smaller sample sizes (Hair et al., 2011). Given that this study had a relatively modest sample size of 269 participants, we opted for the PLS method instead of other SEM techniques.

### 4.1 Measurement model

#### 4.1.1 Internal consistency

The internal consistency reliability of the constructs was evaluated using Cronbach’s Alpha (*α*) and composite reliability (CR). Both measures yielded values between 0.905 and 0.976, surpassing the recommended threshold of 0.7 (see Table 2), thereby confirming strong reliability across all constructs (Sarstedt et al., 2021).

**Table 2:** Outer loadings, Cronbach’s alpha, CR and AVE.

Constructs	Items	Outer loadings	Cronbach’s alpha (*α*)	Composite reliability (CR)	Average variance extracted (AVE)	R-square
SC	SC1	0.968	0.964	0.964	0.933	
	SC2	0.974				
	SC3	0.956				
SCO	SCO1	0.894	0.957	0.957	0.887	0.557
	SCO2	0.970				
	SCO3	0.960				
	SCO4	0.942				
SM	SM1	0.860	0.905	0.908	0.84	0.512
	SM2	0.950				
	SM3	0.938				
SP	SP1	0.853	0.924	0.928	0.768	0.627
	SP2	0.879				
	SP3	0.886				
	SP4	0.922				
	SP5	0.840				
CWS	CWS1	0.975	0.941	0.942	0.895	
	CWS2	0.963				
	CWS3	0.899				
POSP	POSP1	0.955	0.957	0.958	0.887	
	POSP2	0.961				
	POSP3	0.916				
	POSP4	0.936				
AO	AO1	0.955	0.976	0.976	0.932	0.442
	AO2	0.973				
	AO3	0.973				
	AO4	0.961				

SC, safety climate; SCO, safety compliance; SM, safety motivation; SP, safety participation; CWS, co-worker support; POSP, psychological ownership for safety promotion; AO, anticipation orientation.

#### 4.1.2 Convergent validity

To assess the convergent validity of the constructs, outer loadings and the Average Variance Extracted (AVE) were examined (Sarstedt et al., 2021). All outer loadings were found to be equal to or greater than 0.7, and the AVE values were greater than 0.5 (see Table 2). These results indicate that the convergent validity of the constructs was confirmed (Hair et al., 2012).

#### 4.1.3 Discriminant validity

Discriminant validity was evaluated using both the Fornell-Larcker criteria (see Table 3) and the heterotrait-monotrait ratio (HTMT) (see Table 4) (Henseler et al., 2015). According to Table 3, all values in bold along the diagonal (the square root of the AVEs) were greater than the corresponding inter-construct correlations, thus meeting the Fornell-Larcker criteria (Sarstedt et al., 2021).

**Table 3:** Discriminant validity assessment using the Fornell-Larcker test.

Construct	CWS	POSP	AO	SC	SCO	SM	SP
CWS	**0.946**						
POSP	−0.683	**0.942**					
AO	−0.511	0.613	**0.966**				
SC	−0.299	0.359	0.456	**0.966**			
SCO	−0.269	0.330	0.596	0.553	**0.942**		
SM	−0.207	0.287	0.421	0.716	0.631	**0.917**	
SP	−0.549	0.646	0.716	0.446	0.637	0.416	**0.876**

SC, safety climate; SCO, safety compliance; SM, safety motivation; SP, safety participation; CWS, co-worker support; POSP, psychological ownership for safety promotion; AO, anticipation orientation. The bold values in this table represent the square roots of the Average Variance Extracted (AVE) for each variable.

**Table 4:** Discriminant validity assessment using the HTMT test.

Construct	CWS	POSP	AO	SC	SCO	SM	SP
CWS							
POSP	0.719						
AO	0.533	0.634					
SC	0.314	0.373	0.470				
SCO	0.284	0.345	0.616	0.575			
SM	0.214	0.298	0.439	0.762	0.676		
SP	0.586	0.682	0.750	0.473	0.682	0.446	

SC, safety climate; SCO, safety compliance; SM, safety motivation; SP, safety participation; CWS, co-worker support; POSP, psychological ownership for safety promotion; AO, anticipation orientation.

As shown in Table 4, all values in the HTMT matrix are below 0.90(Henseler et al., 2015), indicating that the HTMT criteria are satisfied. Based on this, it can be concluded that both the Fornell-Larcker and HTMT assessments provide strong evidence supporting the discriminant validity of the constructs in the proposed model.

### 4.2 Common method bias

To decrease the potential of common method bias (CMB), first, we adjusted the order of variables on the questionnaire to reduce the respondents’ predictions, and second, we told the respondents that their answers would not be judged. We also employed the full collinearity variance inflation factors (VIFs) test suggested by Kock (2017) to detect common method bias (CMB). According to the author, when factor-based PLS-SEM algorithms are used, a VIF threshold of 3.3 should be applied for CMB tests, while a threshold of 5 is appropriate for algorithms that account for measurement error. The results showed that the full collinearity VIF scores ranged from 1.000 to 1.579. Clearly, all values are below 3.3, indicating that the proposed research model is likely free from CMB. Additionally, following the approach proposed by Podsakoff et al. (2003), Harman’s one-factor test was performed to assess common method bias. The analysis revealed two factors, which together accounted for 51.952% of the variance. The first factor contributed 41.782%, indicating that no single factor explained the majority of the variance. Therefore, we concluded that common method bias did not pose a significant issue in our study.

### 4.3 Structural model

The study employs the standardized root mean square residual (SRMR) to evaluate the overall model fit. The SRMR value was found to be 0.073, which is below the recommended threshold of 0.08 (Hu and Bentler, 1999). This suggests that the model has an acceptable level of explanatory power and fits within a reasonable range. To provide a more comprehensive assessment of model adequacy as recommended by Sarstedt et al., 2021, additional model-fit indices were examined. The normed fit index (NFI) value was 0.923, exceeding the threshold of 0.90 (Bentler, 1990). Furthermore, the structural or inner model demonstrates a causal relationship between exogenous and endogenous constructs (Sarstedt et al., 2021), assessed through explanatory power (R^2^) and predictive relevance (*Q*^2^). The adjusted R^2^ values for Anticipation Orientation (AO), Safety Compliance (SCO), Safety Motivation (SM), and Safety Participation (SP) were 0.442, 0.557, 0.512, and 0.627, respectively (Table 2), supporting the model’s explanatory power (Sarstedt et al., 2021). Additionally, *Q*^2^ values, calculated using the blindfolding procedure, were 0.407 for AO, 0.467 for SP, 0.419 for SM, and 0.476 for Safety Compliance (SCO). All *Q*^2^ values exceeded zero, indicating that the model has substantial relevance for the dependent constructs (Sarstedt et al., 2021).

**Figure 2:**
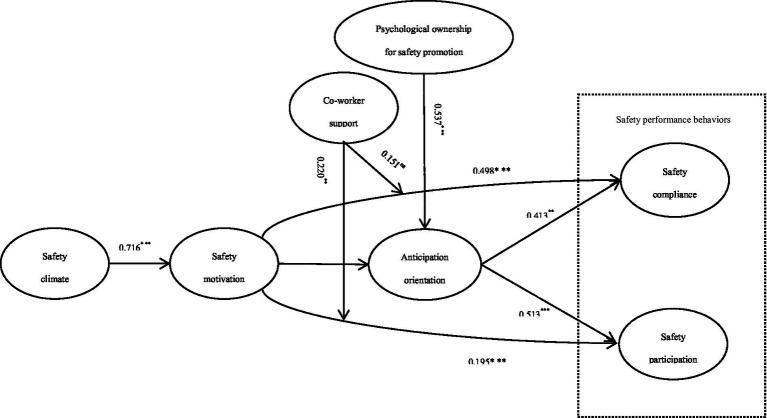
Result of PLS analysis. ns, non-significant, ***p < 0.001, **p < 0.01, *p < 0.05.

The structural model was evaluated through path coefficient (*β*) analysis with corresponding statistical significance indicators, including t-values, *p*-values, and bias-corrected 95% confidence intervals. To ensure robustness, hypotheses were tested using 5,000 bootstrap subsamples. Results of the partial least squares (PLS) analysis are presented in Figure 2 and Table 5. For moderation analysis, we adopted the two-stage approach proposed by Henseler et al. (2015), employing standardized product term generation with automatic weighting. A hypothesis was considered statistically supported if two criteria were met: (1) a *p*-value < 0.05, and (2) exclusion of zero from the bias-corrected 95% confidence interval. Significance levels were categorized as follows: ***p < 0.001, **p < 0.01, *p < 0.05, and ns (non-significant).

**Table 5:** The results of hypothesis testing.

Hypothesis	Path	*β*	*t*	*p*	95% CIs	Support
*H1*	SC → SM	0.716	11.811	0.000* **	[0.588, 0.823]	Yes
*H2a*	SM → SCO	0.498	5.170	0.000* **	[0.333, 0.713]	Yes
*H2b*	SM → SP	0.195	2.651	0.008* *	[0.046, 0.333]	Yes
*H3a*	CWS * SM → SCO	0.151	1.665	0.096 ^ns^	[−0.052, 0.303]	No
*H3b*	CWS * SM → SP	0.220	2.898	0.004* *	[0.053, 0.345]	Yes
*H4a*	SM → AO → SCO	0.110	2.354	0.019*	[0.028, 0.204]	Yes
*H4b*	SM → AO → SP	0.137	2.821	0.005* *	[0.055, 0.244]	Yes
*H5*	POSP → AO	0.537	7.174	0.000* **	[0.391, 0.678]	Yes

*β*, path coefficient; 95% CIs, 95% confidence intervals bias-corrected through bootstrapping; ns, non-significant. * ** *p* < 0.001, * * *p* < 0.01, * *p* < 0.05.SC, safety climate, SCO, safety compliance, SM, safety motivation, SP, safety participation, CWS, co-worker support, POSP, psychological ownership for safety promotion, AO, anticipation orientation.

Our results are as follows. First, the path coefficients of SC → SM (*β* = 0.716, *p* = 0.000 < 0.05, *f*^2^ = 1.050, 95% CIs = [0.588, 0.823] not including 0) is positive and significant. Thus, *H1* is supported.

Second, the path coefficient of SM → SCO (*β* = 0.498, *p* = 0.000 < 0.001, *f* ^2^ = 0.440, 95% CIs = [0.333, 0.713] not including 0) is positive and significant, and the path coefficient of SM → SP (*β* = 0.195, *p* = 0.008 < 0.01, *f* ^2^ = 0.080, 95% CIs = [0.046, 0.333] not including 0) is positive and significant. Thus, *H2a* and *H2b* are supported.

Third, the path coefficient of SM → AO (*β* = 0.267, *p* = 0.004 < 0.01, *f* ^2^ = 0.117, 95% CIs = [0.102, 0.461] not including 0) is positive and significant, and the path coefficient of AO → SCO (*β* = 0.413, *p* = 0.001 < 0.01, *f* ^2^ = 0.244, 95% CIs = [0.143, 0.603] not including 0) is positive and significant, and the positive mediating effect of anticipation orientation on the relationship between safety motivation and safety compliance (SM → AO → SCO) (*β* = − 0.032, *p* = 0.019 < 0.05, 95% CIs = [0.028, 0.204] not including 0) is significant. In addition, the path coefficient of AO → SP (*β* = 0.513, *p* = 0.000 < 0.001, *f* ^2^ = 0.446, 95% CIs = [0.336, 0.692] not including 0) is positive and significant, and the positive mediating effect of anticipation orientation on the relationship between safety motivation and safety participation (SM → AO → SP) (*β* = 0.137, *p* = 0.005 < 0.01, 95% CIs = [0.055, 0.244] not including 0) is significant. So *H4a* and *H4b* are supported. The path coefficients of POSP → AO (*β* = 0.537, *p* = 0.000 < 0.01, ^*f*2^ = 0.473, 95% CIs = [0.391, 0.678] not including 0) is positive and significant. Thus, *H5* is supported.

**Figure 3:**
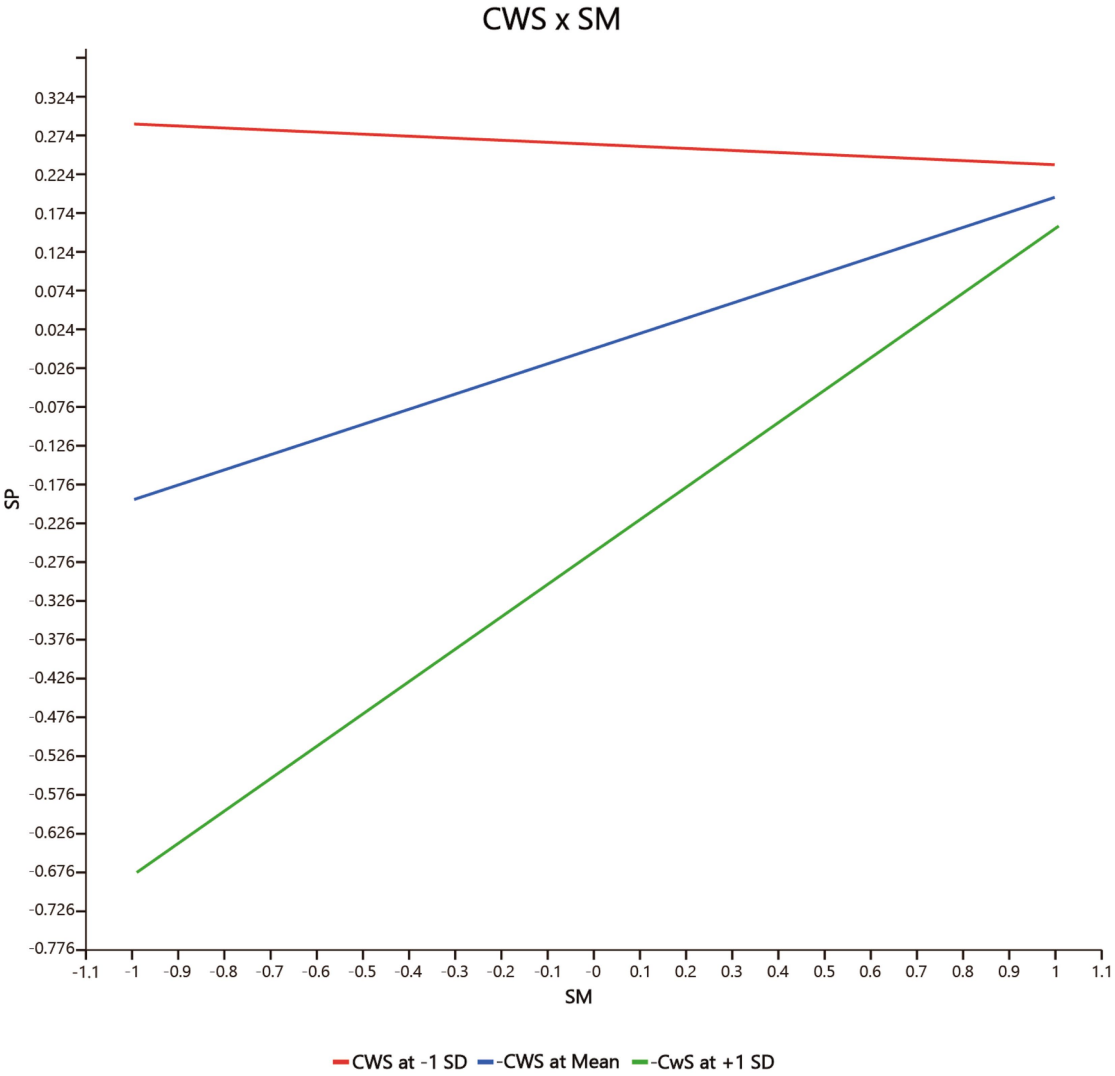
The moderating effect of CWS on the positive relationship between SM and SP. CWS, co-worker support, SM, safety motivation, SP, safety participation.

**Figure 4:**
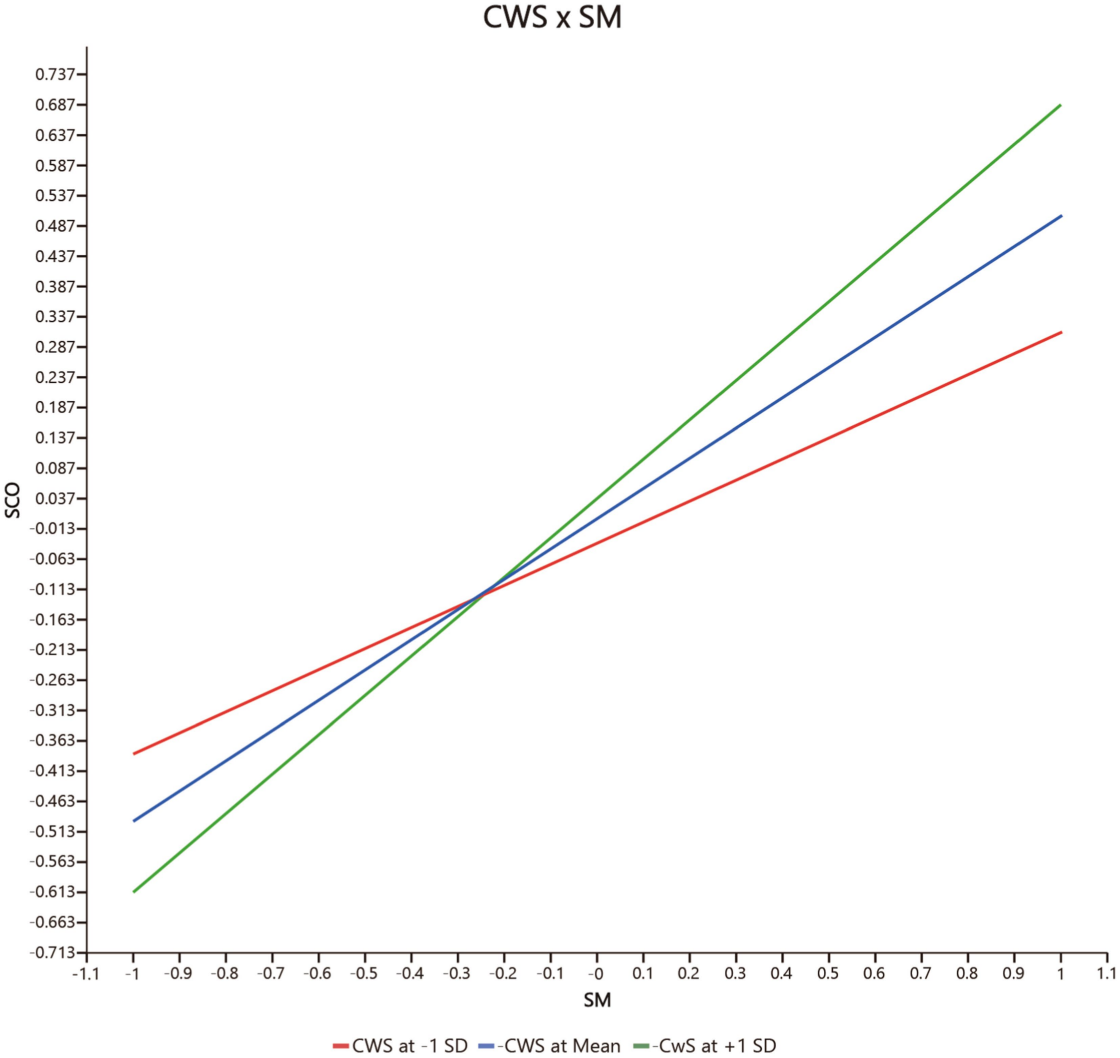
The moderating effect of CWS on the positive relationship between SM and SCO. CWS, co-worker support, SM, safety motivation, SCO, safety compliance.

Fourth, the moderating effect of co-worker support (CWS) on the relationship between safety motivation and safety participation (CWS * SM → SP) (*β* = 0.220, p = 0.004 < 0.01, *f* ^2^ = 0.141, 95% CIs = [0.053, 0.345] not including 0) is significantly positive, but co-worker support has no significant moderating effect on the relationship between safety motivation and safety compliance (CWS * SM → SCO) (*β* = 0.151, *p* = 0.096 > 0.05, *f* ^2^ = 0.055, 95% CIs = [−0.052, 0.303] including 0). Hence *H3b* but not *H3a* is supported. As shown in Figure 3 (CWS * SM → SP) and Figure 4 (CWS * SM → SCO), co-worker support has a greater effect on safety participation when co-worker support is higher, but co-worker support does not significantly influence the relationship between safety motivation and safety compliance.

## 5 Discussion of the findings

### 5.1 Main findings

First, our findings validate Hypothesis 1, revealing a significant positive correlation between safety climate and safety motivation, with safety climate accounting for a substantial share of the variance in the latter. This finding is corroborated by recent studies in healthcare contexts. For example, Jarrar et al. (2023a) showed that poor hospital working conditions may undermine safety behaviors via burnout, underscoring the protective role of a supportive safety climate in maintaining motivation. Similarly, Al-Bsheish et al. (2022) specifically studied ICU nurses and found that management commitment to safety—a core component of safety climate—directly enhanced safety performance by fostering a climate of respect for safety, particularly in high-risk settings like intensive care units. Kim et al. (2021) found, in a multi-country hospital study, that organizational safety climate is a stronger predictor of safety outcomes in healthcare than in other sectors, emphasizing its heightened relevance to climate-motivation dynamics in high-risk, unpredictable nursing contexts.

Second, our results confirm that safety motivation directly boosts both safety compliance (H2a) and safety participation (H2b). This underscores that motivation-fueled compliance is a key mechanism in high-risk healthcare environments, while the weaker effect on participation suggests voluntary behaviors may need extra contextual support beyond mere motivation. Extending this global perspective, a 2023 multi-country study by Saragih et al. (2021) across 35 nations found that nurse safety motivation significantly associated with both compliance (e.g., infection control adherence) and participation (e.g., safety committee involvement) during the COVID-19 pandemic. In high-risk healthcare settings, nurses’ frequent exposure to threats like infections intensifies their perception of safety protocols as critical, thereby amplifying motivation-driven compliance (Afshar et al., 2021). While safety participation is less directly dependent on motivation, it remains vital for proactive risk reduction (Neal and Griffin, 2006).

Third, our findings show that anticipation orientation acts as a mediator between motivation and behavior (H4a-b). Specifically, motivation indirectly influences both safety compliance and safety participation through anticipation orientation. The concept of anticipation orientation is derived from Curcuruto et al. (2016), who highlighted that individuals with a strong orientation toward anticipating risks are more likely to engage in safety behaviors. Similarly, research in European settings provides corroborating evidence. An investigation within the Spanish healthcare system revealed that anticipation orientation served as a critical cognitive mechanism through which safety climate influences safety participation behaviors among nursing staff (Mantas-Jimenez et al., 2022). Another significant finding from our research is that psychological ownership of safety correlates with anticipation orientation, revealing that psychological ownership substantially accounts for the variance observed in anticipation orientation. This relationship is explained by SCT: psychological safety ownership boosts nurses’ self-efficacy (confidence in executing safety tasks effectively) and outcome expectancy (anticipating positive results from proactive safety actions, e.g., risk reduction leading to fewer incidents) by fostering personal control and accountability over safety. Thus, employees with strong safety ownership are more likely to adopt a proactive, anticipatory safety risk approach. This result aligns with prior research on psychological ownership, such as the study by Novieto (2023), who found that psychological ownership directly boosts self-efficacy and outcome expectations, which in turn drive proactive safety participation in high-risk environments. By integrating psychological ownership into the SCT framework, we clarify how it functions as a critical cognitive factor that amplifies both self-efficacy (“I can do this”) and outcome expectancy (“This action will lead to safety improvements”), thereby systematically shaping safety performance.

Fourth, our results show that co-worker support selectively moderates safety motivation’s effects. It amplifies the motivation-participation relationship *H3b* but *not* motivation-compliance *H3a*. This finding aligns with cross-cultural nursing studies, which suggest that the role of social support varies by context. For instance, Japanese clinical nurses emphasize collective responsibility and team-based safety behaviors, where peer support strongly enhances voluntary participation in safety activities, whereas in more individualistic settings, motivation-compliance linkages may rely more on formal systems than peer influence (Eriksson et al., 2025). The non-significant moderating effect of co-worker support on the motivation-compliance relationship may be attributed to the nature of safety compliance itself, which is often clearly defined by organizational protocols and mandated as a baseline requirement. In such contexts, the effect of individual motivation on compliance may be direct and strong, leaving limited room for social support to exert additional influence. Co-worker support, while beneficial for fostering voluntary participation, may not significantly alter the translation of motivation into compliance behaviors that are already strictly governed by formal systems. This delineation is consistent with the job demands-resources model, which posits that discretionary safety participation is more susceptible to motivational and social resources, whereas compliance, as a core task behavior, is primarily regulated by formal job descriptions and accountability systems (Syed-Yahya et al., 2023). This pattern underscores a fundamental distinction in the psychological pathways through which social support operates: it primarily fosters safety participation by nurturing a shared sense of responsibility and collective efficacy, whereas compliance is more directly sustained by formal organizational systems and individual accountability mechanisms.

### 5.2 Theoretical implications

First, this study rigorously substantiates the direct causal link between safety climate and safety motivation (as validated by Hypothesis 1), yielding substantial theoretical contributions. It effectively validates and strengthens the applicability of SCT in medical safety, demonstrating how environmental factors (safety climate) and personal cognitive factors (safety motivation) interact - this is the core principle of the ternary model of this theory. The findings illuminate key mechanisms driving nurses’ safety motivation, revealing that supportive organizational climates transcend institutional constraints to foster intrinsic motivation, thereby advancing insights into individual-organizational safety dynamics. These results provide an empirical basis for SCT, transforming its construction into a testable framework and mapping future paths - such as investigating the mediating role of self-efficacy in the relationship between climate and motivation - to precisely and clearly refine and expand the theoretical paradigm.

Second, this study robustly validates the direct impact of safety motivation on both safety compliance and safety participation, offering profound theoretical insights. It underscores the pivotal role of intrinsic cognitive factors in shaping safety performance, positioning safety motivation as a critical personal driver within SCT ternary model (personal, behavioral, environmental factors). The findings refine our understanding of motivational outcomes: safety motivation not only ensures adherence to basic safety norms (compliance) but also transcends role expectations to fuel proactive engagement in safety enhancement (participation). By confirming the “safety climate → safety motivation → safety behavior” pathway, this research strengthens the explanatory power of SCT, bridging environmental influences and behavioral outcomes. It provides a theoretical foundation for leveraging motivational interventions to simultaneously elevate foundational and proactive safety performance, emphasizing how positive environmental shaping can catalyze intrinsic motivation to drive holistic safety excellence.

Third, this finding reveals a key internal mechanism, clarifying that safety motivation drives behavior not directly, but by shaping an individual cognitive process. Specifically, high levels of safety motivation foster an anticipation orientation in nurses—a proactive cognitive tendency to anticipate potential risks and prepare safety measures—which in turn acts as a more immediate driver of both safety compliance and participation behaviors. This significantly deepens the application of SCT in the safety domain, illustrating that the path from motivation to behavior within “personal factors” involves a sophisticated cognitive transmission mechanism. It highlights the central role of proactive cognitive processes, shifting the research focus from static motivation levels to dynamic cognitive processing, thereby offering a more precise and comprehensive theoretical explanation of how motivation translates into behavior. In addition, this finding substantiates a novel antecedent of anticipation orientation by identifying psychological ownership for safety as a key driver. It extends SCT by delineating a cognitive pathway from a sense of ownership (“my safety”) to a proactive mindset of risk anticipation. This moves beyond traditional motivation-based models, highlighting that fostering personal responsibility and sense of belonging is crucial for developing the foresight needed for proactive safety behaviors, thereby enriching our understanding of the cognitive origins of safety preparedness.

Fourth, the study reveals that co-worker support moderates the relationship between safety motivation and safety participation but not safety compliance (H3b, H3a). This nuanced finding adds to existing research by suggesting that the role of co-worker support is context-dependent. While Syed-Yahya et al. (2023) study have often generalized the moderating effects of co-worker support across various safety behaviors, our findings highlight that co-worker support plays a more significant role in enhancing voluntary safety behaviors (e.g., safety participation) rather than mandatory compliance behaviors. This theoretical distinction provides deeper insight into how social support systems operate differently in motivating proactive versus reactive safety behaviors, particularly in team-based healthcare environments.

Finally, this study makes a significant contribution to the theoretical understanding of workplace safety behaviors, particularly within healthcare settings, by extending and enriching SCT with a focus on safety motivation, safety climate, and proactive safety behaviors. SCT originally emphasizes the role of cognitive, behavioral, and environmental factors in shaping human behavior. Our findings provide new insights into how these elements interact to influence safety behaviors in high-risk healthcare environments, with particular emphasis on the mediating and moderating factors that were previously under explored. Building on cross-cultural evidence from Jarrar et al. (2020), who demonstrated that adverse events in Malaysia are shaped by organizational and contextual factors (e.g., hospital size, accreditation status), our study reinforces the need to test SCT across diverse healthcare systems to enhance generalizability. This aligns with Jarrar et al.’s call for context-sensitive safety frameworks and strengthens the theoretical robustness of SCT in global healthcare contexts.

### 5.3 Practical implications

The findings from this study offer valuable insights for healthcare organizations seeking to enhance safety performance among nurses. By leveraging these results, administrators can develop evidence-based strategies to improve safety behaviors and mitigate risks in healthcare settings. First, our study substantiates Hypothesis 1, demonstrating a strong positive correlation between safety climate and safety motivation. Practically, healthcare organizations should focus on fostering a robust safety climate by prioritizing safety at all organizational levels, ensuring clear communication of safety policies, and demonstrating management’s commitment to safety. The purpose is to enhance nurses’ intrinsic motivation to engage in safety behaviors, addressing the prevalent issue of low safety motivation, which often results in inadequate safety practices (Péculo-Carrasco et al., 2021). However, some scholars argue that individual factors, such as personality traits, may also significantly influence safety motivation (Ling et al., 2019). While this perspective merits consideration, our findings underscore the pivotal role of organizational climate. Implementing safety climate improvements requires sustained effort from leadership and may face resistance or resource constraints. Organizations should invest in regular safety training and visible management involvement to reinforce this climate effectively.

Second, the results confirm that safety motivation directly increases both safety compliance and safety participation. In practice, healthcare organizations should develop interventions that cultivate nurses’ safety motivation, such as highlighting the personal and professional benefits of safety adherence and recognizing proactive safety efforts. The purpose is to enhance both mandatory and voluntary safety behaviors, tackling the current challenge of inconsistent engagement in safety practices that jeopardizes patient safety. By targeting motivation, organizations can directly improve nurses’ safety performance. Some researchers suggest that external incentives or disciplinary measures primarily drive compliance (Smith, 2017), yet our study emphasizes intrinsic motivation’s critical role. Tailoring interventions to the specific needs and context of the nursing staff is essential, as generic approaches may fail to resonate with diverse teams.

Third, our findings indicate that anticipation orientation mediates the relationship between safety motivation and safety behaviors (H4a–b). Healthcare organizations should encourage nurses to adopt a forward-thinking mindset by anticipating potential risks, achievable through scenario-based training and routine risk assessments. The purpose is to foster proactive safety behaviors, addressing the common problem of reactive safety approaches that miss opportunities for prevention. By enhancing anticipation orientation, nurses can mitigate hazards before they escalate, improving overall safety. Critics may argue that cultivating anticipation orientation is challenging due to difficulties in training and measurement (Mann and Savelsbergh, 2015). Nevertheless, our evidence supports its mediating role, suggesting significant value. Organizations must ensure training programs are practical and context-specific to maximize effectiveness.

Fourth, a key finding is that psychological ownership for safety associates with anticipation orientation. Practically, healthcare organizations should foster a sense of personal accountability among nurses by moving beyond general involvement to implementing structured interventions. Concrete, actionable strategies include: (1) establishing unit-level safety committees co-chaired by nurses to review incidents and design preventive measures; (2) creating “safety innovation grants” that fund nurse-proposed risk solutions; and (3) integrating safety ownership metrics into performance development plans, with recognition for proactive risk identification. The purpose is to enhance proactive safety behaviors, addressing the current issue of passive safety attitudes stemming from a lack of ownership. When nurses feel responsible for safety, they are more likely to anticipate and address risks, strengthening the safety culture. Some contend that hierarchical healthcare structures may impede psychological ownership (Häkkinen et al., 2013). Consideration should be given to empowering nurses through participatory roles, ensuring they have the autonomy and support needed to take ownership.

Finally, our study reveals that co-worker support selectively moderates safety motivation’s effects, amplifying the motivation-participation relationship but not the motivation-compliance relationship. In practice, organizations should promote a supportive work environment through team-based safety initiatives and peer mentoring programs. To operationalize this, we recommend implementing structured peer-support programs, such as: (1) Safety Partnership Systems, which pair experienced nurses with newcomers to jointly conduct risk assessments and share safety insights during shifts; (2) Unit-based Safety Champions, who receive specialized training in proactive hazard identification and peer coaching techniques; and (3) Interdisciplinary Safety Huddles, designed as brief, structured forums for staff to collaboratively problem-solve safety concerns and celebrate participation successes. The purpose is to boost voluntary safety participation, addressing the challenge of limited peer support that often hinders such behaviors. A collaborative culture can enhance the impact of safety motivation on participation. We note that co-worker support may be less relevant for compliance, which is more individually driven. Organizations should balance peer-support initiatives with robust policies to ensure compliance, as over-reliance on peer influence may not suffice for mandatory behaviors.

### 5.4 Limitations and future research

Our study has several limitations that offer opportunities for future research. First, due to the cross-sectional design of this study, causal relationships between the variables cannot be established. We explicitly acknowledge this limitation and recommend that future research employs longitudinal or experimental designs to verify the directional effects and causal pathways proposed in our model. Second, data were collected exclusively from nurses in hospitals in Quanzhou, China. This specific geographic and cultural focus may limit the generalizability of the results to other regions, countries, or healthcare systems where safety practices, organizational cultures, and regulatory environments might differ. Specifically, the applicability of our findings, particularly the mediating role of anticipation orientation and the predictive power of psychological ownership for safety, may be influenced by salient cultural and organizational factors. For instance, in Eastern, more collectivistic cultural contexts (such as China), safety behaviors might be more strongly influenced by collective norms and hierarchical organizational structures. In contrast, in Western, more individualistic settings, individual motivation and personal agency, as emphasized by SCT, might play a more dominant and direct role in shaping safety outcomes. Future studies should replicate this research in varied healthcare settings—such as rural versus urban hospitals, public versus private institutions, and across different countries to systematically examine how cultural dimensions (e.g., individualism–collectivism) and distinct healthcare management paradigms (e.g., patient-safety-centric vs. scale-and-norm-centric systems) affect the proposed relationships —to test the robustness and generalizability of the findings. Third, the study depended entirely on self-reported measures for variables like safety motivation, anticipation orientation, and safety behaviors. This approach is susceptible to biases such as social desirability—where nurses might over-report positive behaviors—or recall bias, potentially skewing the accuracy of the findings. To mitigate biases inherent in self-reported data, future research should integrate objective indicators of safety performance, such as recorded safety incidents, compliance audits, or observational assessments of nurses’ behaviors. Combining these with subjective data could yield a more comprehensive and reliable picture of safety performance. Fourth, While the study examined key variables such as safety climate, co-worker support, and psychological ownership, it did not account for other potentially relevant factors, such as individual differences (e.g., personality traits, years of experience) or broader organizational influences (e.g., leadership styles, workload). Additionally, critical contextual factors like work hours, shift patterns and quality of shift handovers—which have been empirically linked to nurses’ safety outcomes and care quality (Jarrar et al., 2023b; Jarrar et al., 2019; Jarrar et al., 2018)—were not measured. For instance, long duty hours and extended shifts may exacerbate fatigue, indirectly undermining safety motivation and compliance. Similarly, variations in handover quality could affect the accuracy of risk anticipation and the execution of safe practices. These unresolved variables may be important factors in shaping safety performance, particularly in high-pressure environments, such as ICUs, or during public health crises. Finally, the study did not investigate the possibility of bidirectional relationships between variables. For instance, engaging in safety behaviors might enhance safety motivation or perceptions of safety climate, creating a feedback loop that was not captured. This limits the understanding of the dynamic interplay among these factors. Future research could investigate potential reverse causality or reciprocal relationships. In summary, building upon the acknowledged limitations, promising avenues for future research include employing longitudinal or experimental designs to ascertain causality and model dynamics over time, expanding the geographical and cultural scope to validate the framework’s generalizability, integrating multi-source and objective data to mitigate methodological biases, and incorporating critical contextual variables—particularly leadership styles and emotional intelligence, and clinical process factors such as shift handover quality—to elucidate their moderating or mediating roles within the safety psychology model. Pursuing these directions will significantly advance a more comprehensive and robust understanding of the complex mechanisms underlying safety performance.

## 6 Conclusion

This study contributes to the understanding of safety performance behaviors among hospital nurses by applying Social Cognitive Theory (SCT) as a framework to analyze how safety climate, safety motivation, anticipation orientation, and co-worker support influence safety compliance and safety participation. First, the study confirms that safety climate is significantly positively correlated with safety motivation, aligning with existing literature and demonstrating that organizational factors play a crucial role in shaping nurses’ safety-related behaviors. The role of safety motivation in driving both safety compliance (adherence to safety protocols) and safety participation (voluntary safety initiatives) is also supported, highlighting the importance of fostering intrinsic motivation among healthcare workers to improve safety practices. Moreover, anticipation orientation serves as a key mediator in the relationship between safety motivation and safety behaviors, suggesting that nurses who anticipate potential safety risks are more likely to engage in proactive safety actions. This is a critical finding for healthcare environments, where anticipating and mitigating risks can prevent adverse outcomes. Additionally, the study identifies that psychological ownership for safety strongly associates with anticipation orientation, further emphasizing the importance of fostering a sense of responsibility among nurses. Finally, the moderating effect of co-worker support demonstrates that peer support enhances safety participation but does not significantly influence safety compliance. This suggests that fostering a collaborative and supportive environment is particularly effective for encouraging voluntary safety behaviors in healthcare settings.

## Data Availability

The original contributions presented in the study are included in the article/supplementary material, further inquiries can be directed to the corresponding author.

## Footnotes

1 www.wjx.cn.

## Appendix A

**Table A1:** Constructs and associated items.

Constructs	Items	Source
Safety climate	(SC1) Management places a high priority on health and safety in nursing workplaces.	Neal and Griffin, (2006)
	(SC2) Management places nursing safety at a high priority.	
	(SC3) Management considers nursing safety to be of great importance.	
Safety motivation	(SM1) I believe it is worth my effort to enhance the personal safety of my patients in my work.	Neal and Griffin, (2006)
	(SM2) I believe it is important to maintain nursing safety at all times.	
	(SM3) I believe that reducing the risk of accidents and incidents in nursing workplaces is of utmost importance.	
Anticipation orientation	(AO1) Even before risk situations actually occur, I ponder various potential risk scenarios to ensure safety.	Curcuruto et al. (2016)
	(AO2) I anticipate and ensure that the future safety condition of my team is clear and favorable.	
	(AO3) I examine situations from multiple safety perspectives to find the most suitable solution.	
	(AO4) When foreseeing risks or safety issues, I consider possible alternative plans.	
Safety compliance	(SCO1) I utilize all necessary safety equipment to complete my work.	Vinodkumar and Bhasi (2010)
	(SCO2) I perform my work in a safe manner.	
	(SCO3) I adhere to the correct safety rules and procedures when performing my work.	
	(SCO4) I ensure the highest level of safety when performing my work.	
Safety participation	(SP1) When my colleagues are working under dangerous or hazardous conditions, I will assist them.	Vinodkumar and Bhasi (2010)
	(SP2) If I notice any safety-related issues within my organization, I always bring them to the attention of management. I will go the extra mile to improve safety in the workplace.	
	(SP3) I voluntarily take on tasks or activities that contribute to improving safety in the workplace.	
	(SP4) I encourage my colleagues to work safely.	
Co-worker support	(CWS1) My colleagues will go the extra mile to make my work life easier.	Leung et al., (2016)
	(CWS2) My colleagues have gone the extra mile to make my work life safer.	
	(CWS3) When difficult situations arise at work, I can rely on my colleagues to provide assistance.	
Psychological ownership for safety promotion	(POSP1) I personally participate in promoting nursing safety activities.	Curcuruto et al. (2016)
	(POSP2) I personally take interest in and drive the proposal of nursing safety initiatives.	
	(POSP3) I should be personally open to embracing and trying new nursing safety management methods.	
	(POSP4) I personally dedicate myself to the implementation of nursing safety plans.	
